# Semi-Automated Image Analysis for the Assessment of Megafaunal Densities at the Arctic Deep-Sea Observatory HAUSGARTEN

**DOI:** 10.1371/journal.pone.0038179

**Published:** 2012-06-05

**Authors:** Timm Schoening, Melanie Bergmann, Jörg Ontrup, James Taylor, Jennifer Dannheim, Julian Gutt, Autun Purser, Tim W. Nattkemper

**Affiliations:** 1 Biodata Mining Group, Faculty of Technology, Bielefeld University, Bielefeld, Germany; 2 HGF-MPG Group for Deep-Sea Ecology and Technology, Alfred Wegener Institute for Polar and Marine Research, Bremerhaven, Germany; 3 University of Glasgow, Glasgow, United Kingdom; 4 School of Engineering and Science, Jacobs University, Bremen, Germany; Utrecht University, Netherlands

## Abstract

Megafauna play an important role in benthic ecosystem function and are sensitive indicators of environmental change. Non-invasive monitoring of benthic communities can be accomplished by seafloor imaging. However, manual quantification of megafauna in images is labor-intensive and therefore, this organism size class is often neglected in ecosystem studies. Automated image analysis has been proposed as a possible approach to such analysis, but the heterogeneity of megafaunal communities poses a non-trivial challenge for such automated techniques. Here, the potential of a generalized object detection architecture, referred to as iSIS (intelligent Screening of underwater Image Sequences), for the quantification of a heterogenous group of megafauna taxa is investigated. The iSIS system is tuned for a particular image sequence (i.e. a transect) using a small subset of the images, in which megafauna taxa positions were previously marked by an expert. To investigate the potential of iSIS and compare its results with those obtained from human experts, a group of eight different taxa from one camera transect of seafloor images taken at the Arctic deep-sea observatory HAUSGARTEN is used. The results show that inter- and intra-observer agreements of human experts exhibit considerable variation between the species, with a similar degree of variation apparent in the automatically derived results obtained by iSIS. Whilst some taxa (e. g. *Bathycrinus* stalks, *Kolga hyalina*, small white sea anemone) were well detected by iSIS (i. e. overall Sensitivity: 87%, overall Positive Predictive Value: 67%), some taxa such as the small sea cucumber *Elpidia heckeri* remain challenging, for both human observers and iSIS.

## Introduction

Despite recent advances in technology and increased efforts to “Census the Marine life”, the deep ocean floor remains the largest and yet least explored ecosystem on Earth [1]. Deep benthic communities are characterized by a high species diversity, which reflects a much larger regional pool of species than in shallow waters [2], constituting a pool of transient potential immigrants to other areas [3]. Megafauna play an important role in benthic ecosystems and contribute significantly to benthic biomass [4]–[6], particularly in the Arctic [7]. Benthic megafauna are defined as the group of organisms inhabiting the sediment-water interface, exceeding 1 cm diameter [8], [9]. Megafaunal organisms increase habitat heterogeneity as they create pits, mounds, tracks and traces in the sediment. Erect biota, such as sponges, bryozoans and coral, increase three dimensional habitat complexity and provide shelter from predation [10], [11]. Megafauna can therefore increase the diversity of smaller sediment-dwelling biota in otherwise largely homogenous soft-bottom environments of the deep-sea [12]–[14]. In addition, megafaunal predators control the population dynamics of their prey and are thus important in determining benthic food webs and community structure [15]–[19]. They also contribute considerably to benthic respiration and affect the physical and biogeochemical micro-scale environment [20]–[26]. It is also important to note that deep-sea benthic megafauna sequester carbon through the continuous redistribution of organic matter, oxygen and other nutrients within surficial sediments [23], [27].

**Figure 1:**
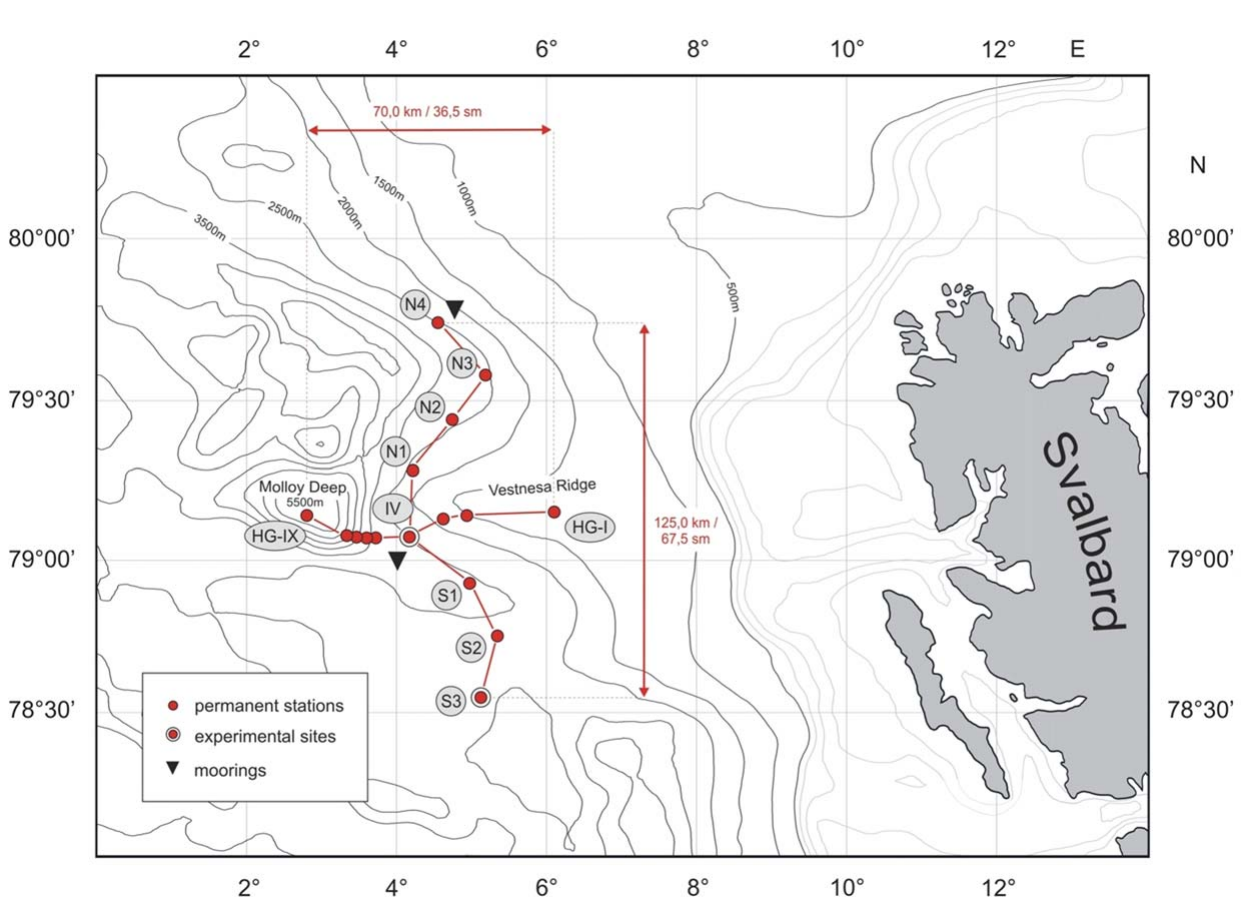
Map of the HAUSGARTEN Observatory. The main sampling station (HAUSGARTEN IV) is located at the intersection of the red lines.

While time series data on megafaunal dynamics over longer scales are still scarce [12], [28]–[31], multi-year time-series studies from the Porcupine Abyssal Plain and the northeast Pacific have attributed megafaunal changes to environmental and climate variation [32], [33]. To date, most studies on megafaunal assemblages in the Arctic represent single snapshots in time, scattered over different basins [7], [34]–[43]. Although such studies provide important biogeographic information, there is currently a serious gap in the knowledge of the temporal dynamics of megafaunal assemblages from these northern latitudes over longer time spans. The HAUSGARTEN observatory [44], established in 1999, represents an important step forward in temporal investigation of the polar region, with large volumes of data collected from the observatory on a regular basis, consisting of both oceanographic data and repeated video and still image collection from a number of fixed survey transects.

**Figure 2:**
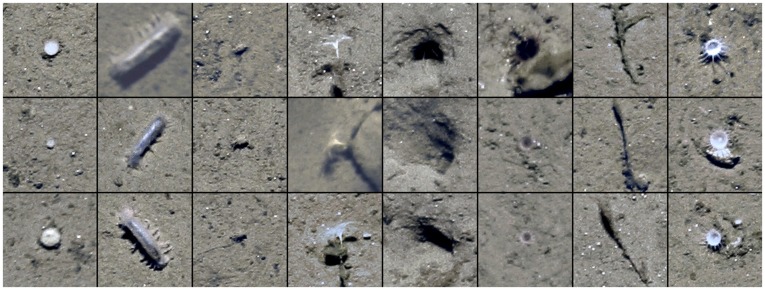
Three samples of each of the eight taxa used for detection. From left to right: small white sponge, *Kolga hyalina*, *Elpidia heckeri*, *Bathycrinus carpenterii*, burrow hole, purple anemone, *Bathycrinus* stalk, small white sea anemone.

Conventionally, megafaunal assemblages are investigated by bottom trawls [45], [46]. However, such gears have low and/or variable catch efficiencies for different organisms [47], [48] and are invasive. In recent years, towed camera systems have become a key method to determine the density and distribution of deep-sea megafauna [29], [40], [42], [49]–[52]. Although visual surveys are limited to species that are large, epibenthic and non-evasive, they enable the study of the seafloor on a range of scales from cm to kilometers without disturbing habitats [53], [54]. Large scale analysis is important, as deep-sea megafauna species are often characterized by rare or aggregated occurrence [43], [55]. Furthermore, this method allows repeated observations of defined tracks, both minimizing the noise produced by spatial variation and allowing time series analysis. Inevitably, the application of imaging techniques generates large quantities of digital image material. Particularly large volumes of footage accumulate in the archives of institutions that run modern remotely operated and autonomous underwater vehicles. The analysis of these images constitutes a bottleneck, since the evaluation of one image with a footprint of 3–4 m^2^, can take 30–60 min or longer, requires training, is subjective and potentially error-prone [56]. Indeed, similar taxonomic classification tasks yielded human consistencies as low as 67–83% (intra-observer) and ≤43% (inter-observer) [56], [57]. To solve this bottleneck problem, computational approaches for taxon detection and classification have been proposed in different contexts. Until now, a number of these are restricted to controlled environments [58], [59], the detection of manufactured objects [60]–[62] or designed to work specifically in the water column [58], [63] where no sediment (i. e. background) has to be distinguished from the taxa investigated. In the majority of published cases, a single taxon or a group of similar taxa [56], [63]–[68] is studied and taxon-customized features are utilized. In other studies, whole images are classified [69] or seafloor images are segmented and each segment is classified automatically afterwards [70]–[73].

**Figure 3:**
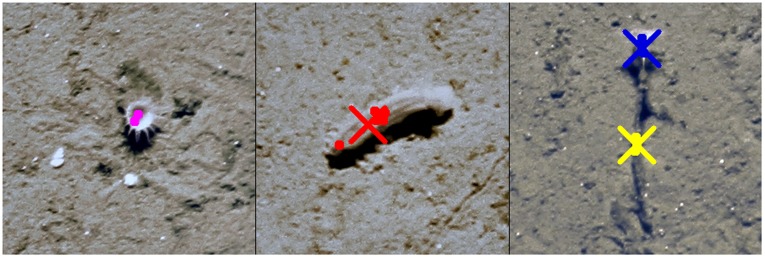
The combination of human labels to gold standard labels. The left image shows a small white sea anemone with two human labels (as circles) which is not enough to create a gold standard label as a supporter count of 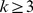 was required (see text for details). The image in the middle shows a *Kolga hyalina* labeled by 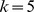 experts and its resulting gold standard label in between (as a cross). The right image shows a *Bathycrinus carpenterii* with human labels for the crown (blue) as well as the stalk (yellow). Both human label cliques have 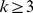 supporter and thus two gold standard labels are created.

**Table 1:** Taxa counts and observer agreements.

			observer agreement			
	Label amounts		inter		intra	
Taxa	Human	Gold std.	average	std-dev.	average	std-dev.
Background	4764	4764	−	−	−	−
*Bathycrinus carpenterii*	2524	503	0.67	0.08	0.80	0.06
*Bathycrinus* stalks	1729	341	0.36	0.08	0.55	0.14
Burrow	5701	1112	0.65	0.04	0.72	0.08
*Caulophacus arcticus*	48	−	0.55	0.15	0.78	0.27
*Caulophacus* debris	131	−	0.44	0.13	0.54	0.24
*Cladorhiza gelida*	59	−	0.43	0.19	0.80	0.21
Purple anemone	498	97	0.68	0.05	0.72	0.07
*Elpidia heckeri*	551	87	0.35	0.09	0.52	0.11
*Gersemia fructicosa*	78	−	0.56	0.17	0.62	0.18
*Kolga hyalina*	172	30	0.97	0.09	0.93	0.07
*Saduria megalura*	67	−	0.53	0.10	0.72	0.14
*Mohnia* spp.	31	−	0.00	0.00	0.10	0.21
Small white sea anemone	2438	457	0.70	0.06	0.79	0.08
Small white sponge	637	94	0.32	0.09	0.55	0.08
Total:	19428	7485				

The taxa with their human and gold standard label amounts. Gold standard labels are computed as the centroid of a group of closely neighbouring human labels of the same taxon. Only groups with 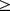
3 human labels were taken into account. The background labels were randomly distributed and were all used as gold labels. Additionally, the inter- and intra-observer agreements are given by average and standard deviation pone.0038179.g001.tif(std-dev.) for the five experts.

To quantify a heterogenous group of megafauna successfully with one system a flexible software approach is needed, which can be applied to taxa exhibiting a variatey of features, such as differing morphologies or colors. The iSIS (**i**ntelligent **S**creening of underwater **I**mage **S**equences) system was developed with such an approach in mind, utilizing a generalized pattern recognition approach for the semi-automated quantification of megafauna in transect data collected at HAUSGARTEN. The approach is referred to as general, since no explicit heuristics were used to design and optimize the algorithmic detection of individual taxa. The taxonomic scope of the system is set to a user defined group of taxa. These groups are defined in the system by a hand-labelled training set of images with marked positions for the taxa. In this way, the user (e. g. a marine biologist) can use her/his primary visual expertise to tune and extend the system without a deeper knowledge of the image-processing algorithms being required. So although the pre-processing and the taxa detection in iSIS runs fully automated, the system is characterized as semi-automatic as the system is trained using these manually identified taxa from within a small image subset of the full transect. In this article we describe the iSIS architecture and present its application to transect data collected at a HAUSGARTEN station. The accuracy of the taxa detection is assessed using a gold standard of taxa positions in 70 images, with this gold standard generated from position labeling of taxa by five experts who evaluated the 70 images manually.

**Figure 4:**
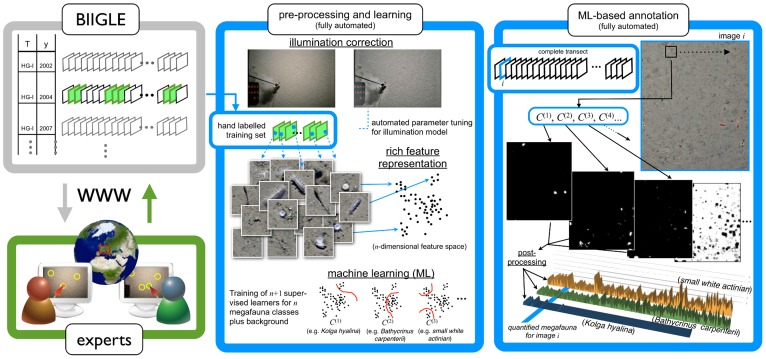
The complete (semi-)automated detection process. Different transects with several thousand images are stored in the BIIGLE online platform (top left). These images can be accessed by experts via the WWW (bottom left). For this experiment, a subset of one transect (marked green on the upper left) was shown to five experts to create a manually labelled training set for a group of pre-defined taxa. Those manual labels were at first used to optimize an image pre-processing for illumination correction (top middle). Afterwards, high dimensional feature vectors were extracted at the label positions to gain a training and test set for SVM optimization (bottom middle). The trained SVMs were then applied pixel-wise to the full field of view, to obtain a confidence value for each pixel and taxon (top right). These confidence values were then post-processed into a classification map, where each pixel is assigned to one taxon which allows taxon counts per image. These taxon counts can then be plotted along the length of the transect (bottom right).

The paper is organized as follows: In the next section, we will first introduce the image data, used in this study. Afterwards, the position labeling study, carried out by the five independent experts is described. The remainder of the section deals with the algorithmic details of the iSIS system. In the results section, the findings of the human position labeling experiment, the pre-processing step and the learning and detection performance of iSIS are presented and discussed.

**Figure 5:**
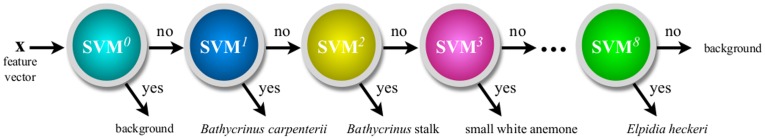
Successive classification with different SVMs. To prevent a time-consuming classification of each feature vector with all SVMs, the SVMs were ordered in a tree structure. The order of SVMs and the confidence thresholds as well as the blob sizes were tuned automatically according to the resulting Sensitivity and Positive Predictive Value.

## Materials and Methods

The deep-sea observatory HAUSGARTEN [44] is located in the eastern Fram Strait west of Svalbard, the only deep-water connection between the Atlantic and Arctic Ocean proper (Figure 1). No specific permits were required for the described field studies as the data was obtained outside national waters. The location of HAUSGARTEN is not privately-owned or protected in any way as it is outside the exclusive economic zone of any nation. To our knowledge our study did not involve any endangered species, and given the remote photographic nature of the data collected, no negative impact on biota was made.

**Table 2:** Training, test and validation performance.

Taxa	Training		Test		Validation		Correlation
	SE	PPV	SE	PPV	SE	PPV	
Background	0.95	0.97	0.91	0.93	−	−	−
*Bathycrinus carpenterii*	1.00	1.00	0.92	0.97	0.74	0.61	0.64
*Bathycrinus* stalks	1.00	1.00	0.86	0.98	0.63	0.38	0.56
Burrow	1.00	1.00	0.98	0.97	0.93	0.50	0.95
Purple anemone	1.00	1.00	0.87	0.98	0.69	0.28	0.28
*Elpidia heckeri*	1.00	1.00	0.82	0.98	0.91	0.04	0.14
*Kolga hyalina*	1.00	1.00	0.53	1.00	1.00	0.88	0.97
Small white sea anemone	1.00	1.00	0.92	0.97	0.86	0.60	0.71
Small white sponge	1.00	1.00	0.73	0.98	0.89	0.43	0.65
**Total**					0.84	0.34	
**Total** excluding*Elpidia heckeri*					0.83	0.50	
**Total** afterre-evaluation					0.87	0.67	

Given are the training, test and validation performance as measured by Sensitivity (SE) and Positive Predictive Value (PPV). The training and test performances are computed with a 4-fold cross validation on the training set. In the validation step, iSIS was applied to the entire images for taxa detection and the detection results were compared to our gold standard 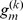 by computing SE and PPV. The performance decreases significantly from the test data to the validation due to an increase in FP. The last row shows SE and PPV results after a careful re-evaluation of the FP (see text for details) yielding our final estimates for iSIS’ SE and PPV. The last column shows the correlation between object counts of the gold standard items and the machine detection result for the full transect.

HAUSGARTEN comprises nine sampling stations along a bathymetric gradient (1200–5500 m). A latitudinal transect crosses at the central HAUSGARTEN station IV, which serves as an experimental area for long-term experiments and measurements [74]–[81]. In 2002, the AWI started regular towed camera observations of the HAUSGARTEN stations during expeditions of the research icebreaker RV Polarstern. To capture images from the seafloor, an “Ocean Floor Observation System” (OFOS) was deployed at different stations with water depths between 1200 and 5500 m (for details see [43]). The OFOS is a towed camera system and its altitude is affected by waves, currents, bottom topography and skill of the winch operator.

**Figure 6:**
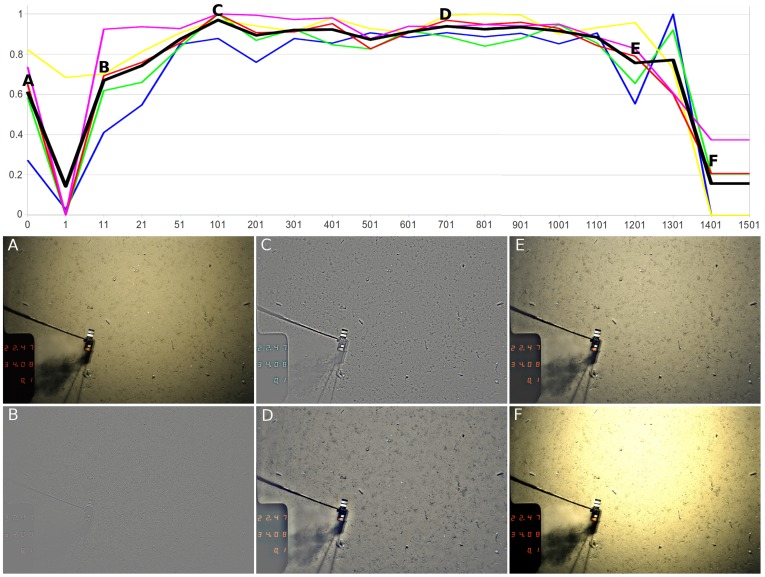
Illustration of the pre-processing. Image A is an original sample taken from the HAUSGARTEN IV transect. B - F show the effect of different kernel sizes *M* for the Gaussian filter. The kernel sizes are as follows: B: *M* = 11, C: *M* = 101, D: *M* = 701, E: *M* = 1101, F: *M* = 1401). The curves show the output of the cluster-indices, plotted against *M*. The first value (*M* = 0) represents the unfiltered image. The curves are as follows: blue: Chalinski-Harabasz, green: Index-I, yellow: Davies-Boudlin, pink: intra-cluster variance, red: inter-cluster variance. The bold, black line is the mean of the five measures. The cluster indices were normalized to the interval [0.1] and show a good correlation, supporting a reasonable selection of the value *M* = 701.

From 2002–2008, more than 45,000 images were taken by an analogue camera, with these images then digitized at a resolution of 3504×2336 pixels. The images were then made accessible in the BIIGLE online platform for browsing and taxa annotation [82]. A number of benthic megafauna experts have acquired BIIGLE accounts in the last two years and to date have labelled 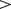
350,000 objects in 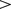
12,000 images. For this study, one transect of intermediate water depth was chosen (HAUSGARTEN IV, 2500 m [31]), which has been successfully visited four times by Polarstern to date (2002,2004,2007,2011). During each campaign, some 700 images were taken. In all images, a field of view of 1500×1800 pixel size at position x = 1800, y = 300 was selected, to exclude the image region covered by the OFOS forerunner weight and the camera time stamp.

**Figure 7:**
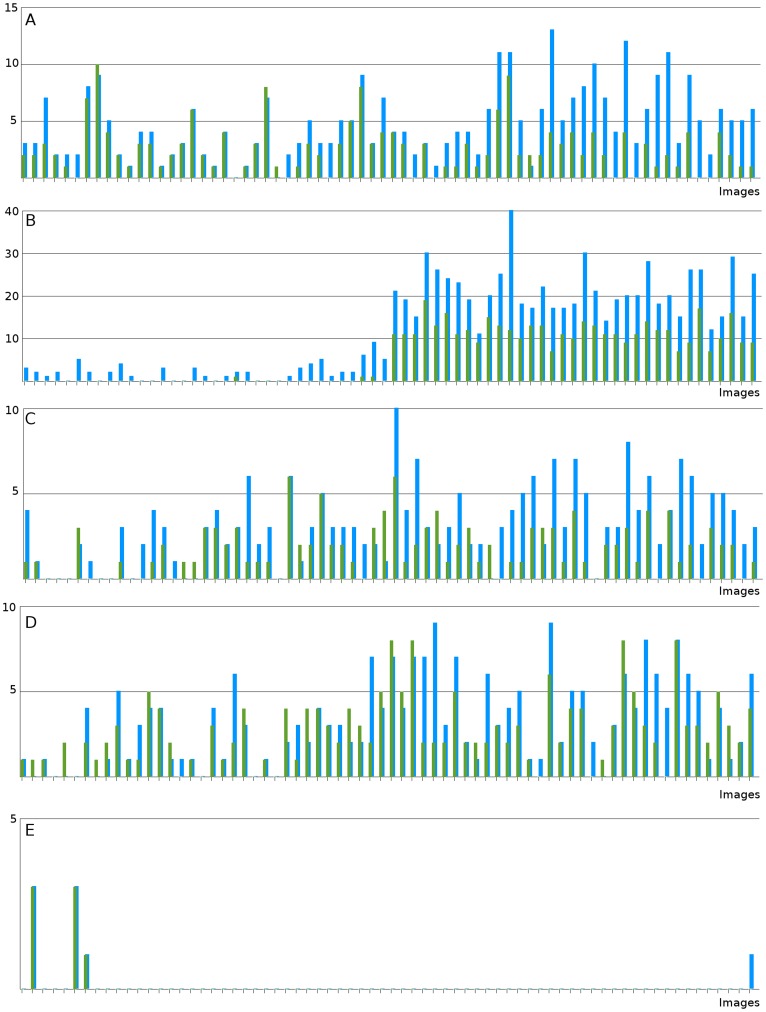
Detection results for five species. From top to bottom: A: small white sea anemone, B: burrow, C: *Bathycrinus* stalk, D: *Bathycrinus carpenterii*, E: *Kolga hyalina*. Each unit on the x-axis represents an image of the transect (i. e. 70 images). The y-axis represents the object counts. Green bars stand for the amount of gold standard objects with 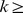
3. Blue bars represent the machine counts. The plots are normalized according to the maximum object count for each taxon individually. The correlation between the gold standard and machine counts are given in Table 2.

**Figure 8:**
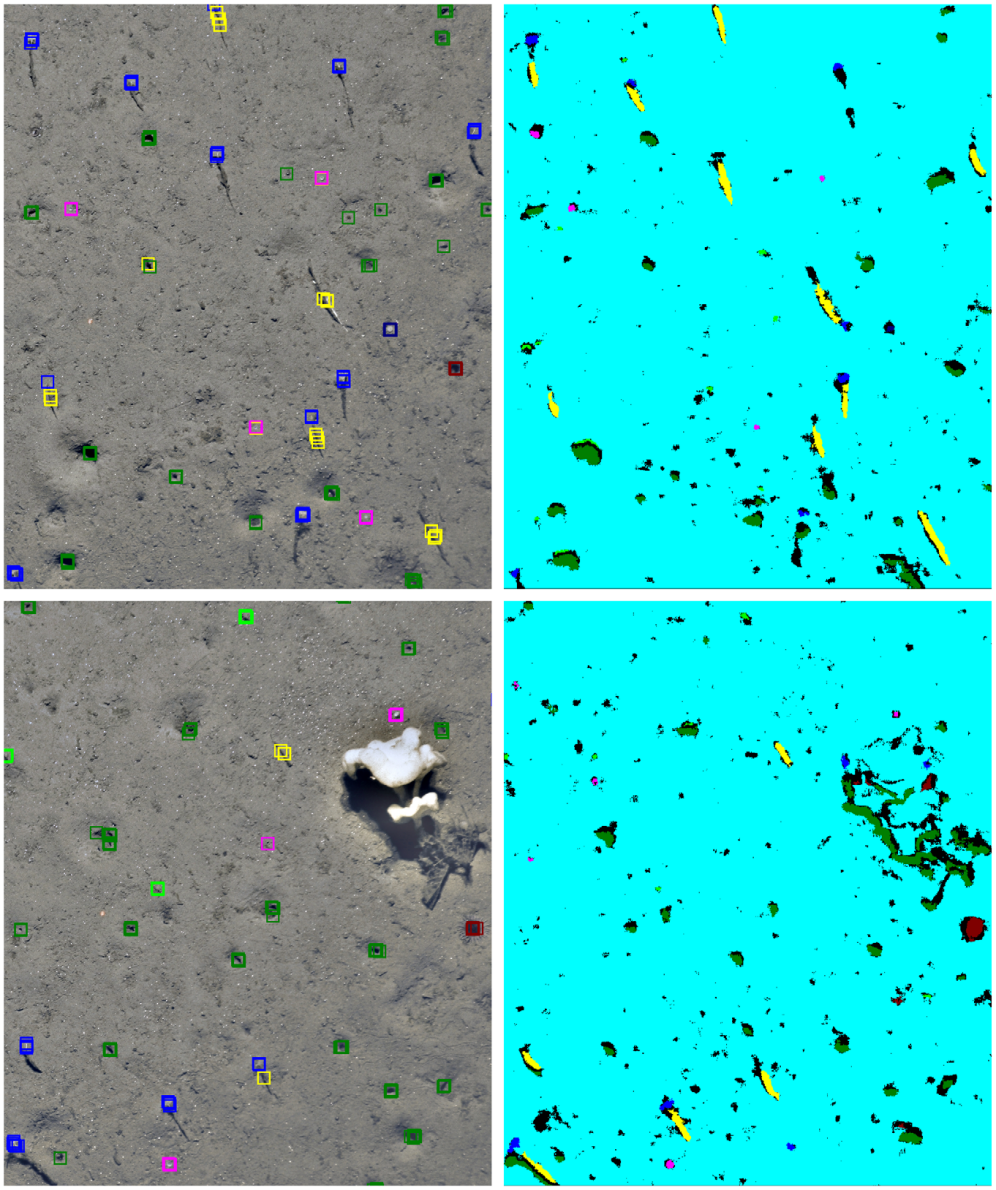
Example of the final classification. On the left we show the original seafloor image with expert labels shown as colored squares. The colors encode taxa: red: *Kolga hyalina*, green: *Elpidia heckeri*, blue: *Bathycrinus carpenterii*, yellow: *Bathycrinus* stalk, pink: small white sea anemone, dark blue: small white sponge, dark red: purple anemone, dark green: Burrow, turquoise: background. On the right we show the images’ classification results. The same color code is used as for the expert positions labels on the left. Black regions were rejected by all SVMs.

The OFOS operator tried to maintain the OFOS at a uniform 1.5 m height above the seafloor, resulting in a real-world footprint of 1.2–8.5 m^2^ per image with an average of 3.77 m^2^ across the entire transect. The OFOS altitude varied throughout the entire transect as the winch operator adapted to bottom topography and sea state resulting in variable lighting conditions, with overexposed images produced when the OFOS was too close to the seafloor, and almost black, poorly illuminated images produced when the OFOS was too distant from the seafloor. Some 10% of the images of a transect showed no signal contrast at all and were excluded from this study. The remaining images showed a decrease in lighting and contrast towards the image corners - a vignette effect.

**Table 3:** Re-evaluation of false positives.

	Expert 1	Expert 2
True positives	26%	35%
Misclassification	9%	12%
Untrained taxa	17%	38%
Background	32%	11%
Unknown	16%	4%

Re-evaluation results of the detected false positives by two authors (MB,TS).

### Human Expert Labeling

The basic idea behind the iSIS architecture is that a general machine learning based object detection system acquires the knowledge of the structural features of objects of interest (here taxa) as well as the non-interesting patterns from a set of image patches showing representative examples of all taxa. The performance of the system can be assessed using a so-called gold standard, created from taxa positions provided by human experts for comparision with the machine produced results. Since we were aware of the inter- and intra-observer agreement problem in human expert labeling tasks, we carried out a position labeling study with five human experts (i.e. the authors M. B., J. T., J. G., A. P. and J. D. ). This study had two aims: firstly, assess the taxon-specific human experts’ inter- and intra-observer agreements across a range of images. The second aim of this study was to allow collection of human expert position labels for use in generating a gold standard for the taxa detection. To carry out the study, a subset of 10% of the 2004 transect (i. e. *N* = 70 images) were shown to five experts. These 70 images were randomly chosen from those with a footprint of 3.5–4.5 m^2^ (i. e. 226 images). The experts were given the task of labelling the positions of all individuals in these images belonging to a set of 14 taxa/seabed features (the sponges *Cladorhiza gelida*, *Caulophacus arcticus*, *Caulophacus* debris, a small white sponge, the soft coral *Gersemia fruticosa*, a small white sea anemone, a purple anemone, the whelk *Mohnia* spp., the isopod *Saduria megalura*, the sea cucumbers *Kolga hyalina* and *Elpidia heckeri*, the sea lily *Bathycrinus carpenterii*, *Bathycrinus* stalks and “burrow hole”). Taxa that gathered 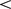
150 labels across the 70 images were excluded from further analysis. Samples of the eight remaining taxa (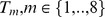, including the category “burrow hole”) are given in Figure 2.

**Table 4:** Classification performance by supporter.

*k*	1		2		3		4		5	
Taxa	SE	PPV	SE	PPV	SE	PPV	SE	PPV	SE	PPV
*Bathycrinus carpenterii*	0.58	0.63	0.68	0.62	0.74	0.61	0.80	0.60	0.85	0.54
*Bathycrinus* stalks	0.51	0.49	0.60	0.45	0.63	0.38	0.64	0.25	0.82	0.10
*Burrow*	0.88	0.53	0.89	0.52	0.93	0.50	0.91	0.46	0.96	0.39
Purple anemone	0.57	0.28	0.65	0.28	0.69	0.28	0.79	0.27	0.90	0.23
*Elpidia heckeri*	0.68	0.05	0.75	0.05	0.91	0.04	0.96	0.03	1.00	0.01
*Kolga hyalina*	1.00	0.88	1.00	0.88	1.00	0.88	1.00	0.88	1.00	0.86
Small white sea anemone	0.71	0.63	0.79	0.62	0.86	0.60	0.91	0.59	0.94	0.53
Small white sponge	0.53	0.48	0.61	0.46	0.89	0.43	0.89	0.29	0.91	0.19
**Total**	0.70	0.37	0.77	0.36	0.84	0.34	0.88	0.30	0.93	0.24
**Total** excluding *Elpidia heckeri*	0.70	0.54	0.77	0.53	0.83	0.50	0.88	0.46	0.93	0.39

Given are the Sensitivity (SE) and Positive Predictive Value (PPV) for all taxa, compared to different supporter values *k* for the gold standard. While the SE is increasing with increasing supporter value, the PPV is performing inversely. This shows, that the automated detection is more likely to find objects with a high observer agreement.

We chose the 2004 transect as it had already been extensively labelled by two of the experts and it was evident that different species, characterized by a variety of structure and color features, occurred in this image series. This species heterogeneity was important to investigate the general applicability of the iSIS system.

The position labeling results of the five experts were compared to determine inter-observer agreements [83]. Observer agreements (OA) were computed for all pairwise combinations of two experts *U* and *V* and their corresponding sets of hand labels 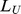 and 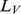 by:
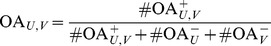
(1)where # means “items in” and 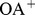 is given as the set of labels contained in both 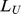 and 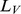:
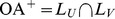
(2)and 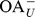 as the set of labels contained only in 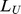:
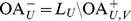
(3)and analogous for 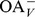.

To measure intra-observer agreements, each expert re-examined 35 images after 14 days. The intra-observer agreements were computed for each expert *U* and her/his hand labels created before (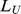) and after the 14 day break (i. e. 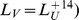) with eq. (1).

To collect a gold standard for the taxon detection, the position labels 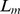 (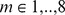) for each taxon 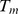, obtained by all five experts within an image were fused to taxon cliques, each clique summarizing the marked positions for one object of a taxon class 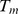. A set of position labels of one taxon 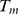 with a pairwise Euclidean distance smaller than a taxon-specific maximum distance 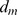 is regarded as a clique. The number of labels in a clique is denoted by *k*, which ranges from 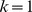, where only one expert (i. e. supporter) found the item, to 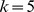, where all experts agree on the occurrence of this item. For each clique, a gold label position 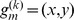 of 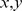-coordinates was computed as the centroid of its supporting clique’s position labels (see Figure 3). The taxon label numbers and observer agreements are given in Table 1.

### The iSIS System

The approach for object detection encompasses three major steps: Pre-processing and feature extraction (Step 1) is necessary to reduce illumination effects and to map image patches to high-dimensional representations in a vector space model, so-called feature vectors. In Step 2, these feature vectors are used to train a machine learning algorithm (Support Vectors Machines (SVMs)), utilizing the human expert position labels. To detect the taxa in one image, the trained SVM classifiers are applied to the feature vectors derived at every pixel within the field of view of each image. The pixel-wise classifications are written to so-called confidence maps. In the final Step 3, these confidence maps are then post-processed to derive positions of possible taxa and a numerical value for the number of taxa in every field of view. An overview of the whole approach is given in Figure 4.

#### Step 1: Feature extraction and pre-processing

To keep the taxon detection as generic as possible, a set of feature descriptors capable of describing arbitrary objects, based primarily on the MPEG7 standard was computed [84], [85]. The MPEG7 standard defines 18 descriptors for different characteristics of digital images, each descriptor comprising an individual number of features. There are five descriptors for color features, three for texture, and ten others, which focus on structure, motion and face detection. Depending on the image domain, some of these are more useful than others (e. g. face descriptors were not used here). The descriptor set consisted of four color descriptors (i. e. Color Structure, Color Layout, Scalable Color, Dominant Color), one texture descriptor (Edge Histogram) as well as an adapted structure descriptor [86].

In principle, the two other texture descriptors specified in the MPEG7 standard would have been useful too, but require a minimum region for extraction (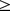
128×128 pixels), which would have added too much background signal in this setup. Those MPEG7 texture descriptors are based on a multi-scale, multi-orientation Gabor Wavelet filtering and describe spatial relationships between Gabor responses as well as dominant responses in the extraction region. To include the principal ability of Gabor Wavelets to describe textural features, the outputs of a modified version of a 3-scale, 5-orientation Gabor bank [87], [88] were added as additional features without regard to their spatial occurence or dominance.

Features were extracted within a frame of 32×32 pixels to create a rich feature representation of 424 dimensions (Figure 4, middle) for a neighborhood around an image pixel.

To correct the lighting conditions of an image 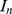 (*n* = 1.*N*), it was filtered with a Gaussian kernel of size *M* and yielded a smoothed image 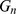. 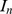 and 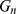 are composed of three color channels *c* (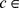 red (R), green (G), blue (B)), here denoted by a superscript (e. g. 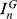 for the green channel of image 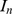). By subtracting 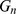 channel-wise from 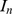, the lightness falloff towards the corners was removed:
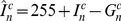
(4)Afterwards, the histograms of each of the 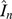 were transformed to gather similar color distributions across the whole transect and thus yielded the image 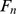 that was then used for feature extraction. 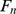 is also a 3-channel image and each of the channels is computed by:
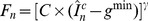
(5)with:
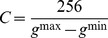
(6)and:
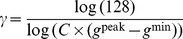
(7)The values 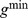, 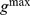 and 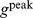 were computed using the gray-scale image 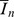:
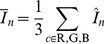
(8)by searching the peak in the histogram of 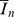 (i. e. the gray value 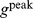 with the highest pixel number in 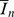). Starting from the peak value, 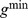/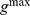 were chosen as the nearest gray values below/above the peak with 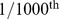 of the peak’s pixel number.

To omit a dispassionate manual tuning of one important parameter in this pre-processing, the Gaussian kernel size *M*, a data driven tuning approach was developed. Feature vectors were computed for each human label position from images pre-processed with different *M* values, ranging from 1 to 1501. Following the standard pattern recognition paradigm, feature vector clusters should identify the taxa. This motivates the selection of that particular *M* value that leads to separated taxa feature clusters, i. e. a crisp cluster structure. To measure the clustering quality for different values of the kernel size *M*, the cluster indices (i. e. Chalinski-Harabasz [89], Index-I [90] and Davies-Boudlin [91]) were computed as well as the intra- and inter-cluster variance. The kernel size *M* leading to the best clustering result was chosen for pre-processing the entire transect.

#### Step 2: Training data and machine learning

For the machine learning step, i. e. teaching the classifiers to distinguish one taxon from other objects and from the background, training sets of feature vectors for each taxon are required. To collect a training set for a taxon 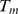, feature vectors were computed for positions 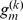 condition to a support count 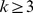. Because of the low numbers of remaining taxon labels, caused by some taxa’s sparse population of the seafloor, the amount of feature vectors were boosted five-fold by computing them at the human label positions as well as at their 4-connected neighbours [92] in two pixel distance. This also adds some variation to the taxa representations. In addition, feature vectors were extracted from randomly distributed positions within each image and with a minimum distance to all human labels within each image. These feature vectors served as representatives of the background class (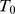).

In pattern recognition, normalization of features is of crucial importance. In the iSIS system, features are grouped according to domains, e. g. all of the 15 Gabor features or the single “number_of_dominant_colors” feature (which belongs to the descriptor Dominant Color [84], [85]) form two domain groups. Feature domain groups are treated individually in the normalization and features values within a group were normalized together to have a mean of 0 and a standard deviation of 1.

After normalization of all domain groups, an individual set of feature vectors 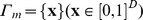 was composed for every taxon 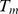, that consisted of 50% positive and 50% negative samples. The positive samples were all feature vectors computed for training set positions of one taxon 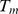. One half of the negative samples consisted of background (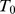) feature vectors. The other half comprised equal amounts of all other taxa 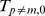. The background feature set 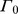 consisted of 50% background samples (positives) and 50% of equal amounts of all other taxa (negatives). Since the abundance of species varied, the size of the feature vector sets 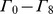 also varied. Using the nine feature vector sets 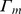, nine classifiers were trained, each one to classify a feature vector as either taxon-positive or negative. iSIS uses SVM classifiers [93] for feature vector training and classification. SVMs are widely used, because of their generalization performance in non-trivial, high-dimensional feature spaces, i. e. their ability to correctly classify previously unseen data. Further advantages are the absence of local minima in their training errors during optimization [94] and the low number of parameters (i. e. two in this case) that have to be tuned.

To train the nine SVMs (one for each taxon and one for the background), an implementation of SVMlight was used [95], wrapped by our own C/C++ machine learning library. A Gaussian kernel was used, and, in a first training step, optimal parameters for the kernel size 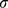 and the SVM penalization parameter *C* were estimated by logarithmic sampling of the parameter space (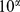, 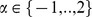 for *C* and 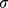, respectively). Small values of *C* indicate low penalization of errors, leading to a better generalization. Small values of 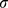 can create SVMs that tend to over-fit the training data, which results in a poor generalization. A 4-fold cross validation [96] was applied to tune the SVM parameters, i. e. three quarters of the feature vector set 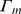 were used as the SVM training set for the SVM of taxon 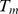 and the 4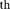 quarter as the test set.

A trained SVM classifies a feature vector either as class-positive or -negative. The classification result for one feature vector can thus be assigned to one out of three groups: 1) True positives (TP) were correctly identified positive samples. 2) False positives (FP) are negative samples, that were falsely classified as positive. 3) False negatives (FN) are positive samples classified as negative. From the counts of these three cases, standard classifier-performance measures (i. e. Sensitivity (SE), Positive Predictive Value (PPV)) were computed for the classification of the training and test sets. SE and PPV are defined as:
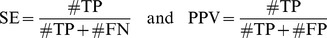
(9)where # means “amount of”. Both measures range between 0 and 1. The PPV of the test set is of special interest in detection tasks, since the highest priority is to minimize the number of false positives in unseen data. After determination of the optimal 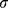 and *C*, the SVM training was repeated for each taxon 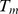 with the full feature vector set 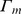. Those SVMs were then used for classification of the full field of view of all images 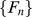 in the transect.

#### Step 3: Post-processing

All SVMs were forced to create a normalized output between 0 and 1 [97], such that a confidence map is created for each species. To gather a quantification of the species at hand, a post-processing was applied to the confidence maps, which consisted of two steps for each taxon 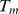. First the confidence maps were binarized with thresholds 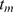. Connected regions (i. e. blobs) in the resulting binary image were then compared to a taxon-specific minimum blob size 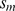. The trained SVMs were organized to a tree (Figure 5), to avoid a time-consuming classification of each pixel by all nine SVMs. Because the average taxon occurrence per image was sparse (i.e. ranging from 0.5 for *Kolga hyalina* up to 16.3 for “burrow hole”), a 5 pixel margin around classified pixels was not used for further classification, to prevent false positives in unusually short distances around detected objects.

For the background confidence map, the binarization threshold was set to 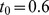 and no blob detection was performed. The other taxa confidence thresholds 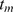, the minimum blob size thresholds 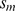 and the order of the SVMs in the classification tree were automatically tuned, analogous to the pre-processing. Similar to the fusion of human label cliques to gold standard labels, a distance threshold was used for each taxon 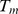 to match the gold standard labels 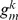 with the detected blob centroids. These assignments were evaluated to classify each blob centroid and each gold standard position as TP, FP or FN. From these quantities, the SE and PPV were computed.

## Results

The human experts showed varying degrees of inter-observer agreement across different taxa, which is a phenomenon well-known from similar visual diagnosis and assessment tasks. An agreement of 97% was found only for the conspicuous sea cucumber *Kolga hyalina* whereas the human detection performance was only 70% for a small white sea anemone and even 35% for the sea cucumber *Elpidia heckeri* and 32% for a small white sponge. While the semi-automatic approach showed a performance at least similarly accurate for the “easy” species (see details below), it produces good, and, above all, re-producible detections (Table 2). The performance for taxa with morphological characteristics close to the resolution limit prevented successful identification by either humans or iSIS. In the following we will summarize the results obtained using the iSIS approach.

### Pre-processing

For the tuning of the Gaussian kernel size *M*, all cluster indices showed similar results: features extracted from unfiltered images were sub-optimal in their ability to create clusters in feature space, while pre-processing with small kernels (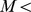
20) reduced the performance even further. The same occurred when using larger kernels (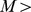
1100), where the cluster measures tended to show poorer results as well. Interestingly, all cluster measures remained relatively stable for kernel sizes between 50 and 1000 with no obvious peak, i. e. no optimum kernel size could be derived. Although the color distributions of differently pre-processed images are diverging (small kernels lead to grayish images with high contrast, large kernels to smoother colors with less local color deformation), the utilized features do not seem to be affected in their capability to form taxon clusters. A kernel size of *M* = 701 was chosen, as the resulting images usually showed good lighting correction and contained only moderate color distortion. Some examples of the pre-processing and the normalized output of the cluster indices are given in Figure 6. The pre-processing takes ca. three minutes per image.

### Machine Learning and Post-Processing

The normalization of the feature vectors and the construction of the training and test sets took less than one minute. The SVM parameters differed for different species. The performances for training and test data are given in Table 2. The first training step to determine the optimal *C* and 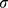 took about five minutes per SVM, which is the same as the time needed for the final SVM trainings together. The post-processing takes less than one minute for all nine SVM outputs combined.

The performance measures for iSIS are shown in Table 2. The classification performance on the training data is displayed as training- and test-error, showing a satisfying learning result for all taxa. In the validation experiment, we applied the trained SVMs to every pixel within the full field of view for a pixel classification and to be able to detect the taxa. We evaluated the classification result using our gold standard 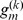, computing SE and PPV for each taxon class individually. The total counts for the gold standard and the machine results are given for each image in Figure 7, the correlation values of these are also given in Table 2. These correlation values give a different view of the results. While the SE and PPV values show the detailed performance at single object level, the correlation measure averages out some mistakes if false positives and false negatives occur within the same field of view. Two examples of the detection result are given in Figure 8.

The final results, as given in Table 2, look unsatisfying at first sight, especially the PPV values for the detection experiment in the entire images, i. e. the validation. A closer look at single FPs leads to the assumption that the false positive counts based on the reference gold standard were incorrect, i. e. many positives found by iSIS, which were not included in the gold standard were actually true positives. All false positives were thus re-analyzed by two of the authors (the authors M. B., T. S. ) to determine, what kind of mistakes happened during the detection. The results of this re-evaluation are given in Table 3. The last row of Table 2 incorporates these numbers and indicates a much better performance (SE: 87%, PPV: 67%). Approximately one third of the false positives were indeed true positives that were not labelled by the experts at all (*k* = 0) or were not included in the gold standard due to a low supporter count (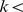
3).

To study the effect of a higher or lower value of *k*, i. e. the effect of a more or less conservative gold standard setting, iSIS was run with values of 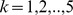. This was done only in the post-processing, so the SVMs were not retrained, which would have affected the detection process. While a low value of *k* resulted in a higher PPV, a high value of *k* resulted in a higher SE. The performance values for different *k* are given in Table 4. The results show that the performance of a semi-automated detection approach is significantly affected by the initial training gold standard. If the main goal is to lose the lowest number of objects, which are prototypal for their species, only gold standard objects with a high supporter value should be used in the analysis.

One particular taxon (*Elpidia heckeri*) could not be detected reliably since its features (color and morphology) could not sufficiently be discerned from the sediment background. Samples of this species cover only a small amount of pixels (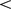
50) and resemble stones in their structural appearance. While the SE of 0.91 is satisfying, the PPV of 0.04 shows, that a vast amount of false positives are detected by the SVM trained for this taxon. The challenges in detecting *Elpidia heckeri* with iSIS reflect the low inter- and intra-observer agreements for this species. Omission of *Elpidia heckeri* from the detection process led to a removal of about half of the total false positives (see 13th row in Table 2).

## Discussion

In our work we have addressed the question how the concepts of pattern recognition and machine learning can be applied to design a data-driven approach to the automation of taxa detection and megafauna quantification in large underwater image collections from camera transects. The most important design principle of this study was to develop a system which would enable a non-computer expert, a typical skilled taxonomist or other user, to adapt the system to new transects and/or for detection of further megafauna species (for instance starfish, which have not been considered here but occur in HAUSGARTEN transect data). Our results show how a gold standard of human labelled taxon positions in a training subset of images can be used to tune pre-processing steps and to train supervised machine learning algorithms (such as SVMs) in pixel classification tasks. Our results for training-, test- and validation errors show that the biggest remaining challenge is to improve the training step, reducing the FPs in the validation and to improve the estimates for the errors on new data, since the contrast between test- and validation error is considerable. We also found two factors to have a negative influence on the PPV estimates. First, the re-evaluation of the FP showed that about one third of the false positives were indeed true positives. Second, another 30–40% of the FP were species that were not included in the SVM training (e. g. *Caulophacus arcticus*, *Caulophacus* debris, *Mohnia* spp., etc.). Thus, including these species in the training data could have the potential to reduce the FPs. We estimate the remaining *true* FPs to be approx. 30% of the original number. These FPs are misclassifications between different taxa or background pixels classified as taxa. Incorporating the additional true positives, the total SE value rises to 0.87, which is only a minor advantage, although the total PPV is then 0.67, which is a major improvement. Assuming optimistically that those species for which iSIS was untrained so far in this study can be identified with similar SE and PPV values as the species thus far studied, the PPV for the dataset as a whole could potentially increase to 0.83. Another strategy to further improve the results would be to omit regions within images from classification, based on the density of detected objects. Parts of images, covered by fauna such as *Caulophacus arcticus* (Figure 8, bottom), create several FP, which are closely distributed and hence distinguishable from other regions as detection results are usually sparse.

Although false positives remain in the iSIS analyzed data, the system can be applied to speed up the quantification of megafauna taxa substantially. A full manual evaluation takes approx. 30–60 minutes per image (and is error prone for many taxa). One way to massively reduce this time would be to first apply iSIS to mark all potential positions of taxa of interest and let a user review the positions (for instance in a guided zoom-in mode) and mark iSIS produced detections as *accept* or *reject*. Such a posterior evaluation of iSIS-detected taxa in an image takes about 1 minute, estimated from our own experience using the BIIGLE system in similar contexts. Without such a re-evaluation, the detection results may overestimate the occurences of taxa and can thus not yet be used for quantitative investigations of transects.

If the system is not trained with key species of the ecosystem, the detection counts may lead to incomplete or incorrect assumptions about habitat processes. Careful consideration of relevant species and suitably large sample sizes of those species are therefore vital for successful application of the iSIS approach.

The number of individual examples of a species required for successful detection may vary across species. An approach to estimate this amount could utilize cluster indices as for the optimization of the kernel size *M* in the image preprocessing. The iSIS system could thus request further labels from the expert if the cluster indices indicate that insufficient amounts of a taxon have been labelled (i.e. the feature representations of this taxon do not yet form clusters in the feature space).

Keeping those prerequisites in mind, iSIS currently allows the collection of taxa positions with reduced effort, which enables researchers to carry out investigations of the taxa densities, their dynamics over time and species co-occurences more efficiently. This could potentially open the large data archives created by far-sighted seafloor observation programmes and give deeper insights into distributions and dynamics of communities of benthic megafauna. The use of iSIS with re-evaluation allowed us to quantify megafaunal densities over the whole HG IV transect for the first time (Fig. 4 bottom right). From this analysis, certain conclusions on species distribution are immediately apparent, such as a patchy occurrence of the small white sea anemone (possibly *Bathyphellia margaritacea*) along the HG IV transect, which is corroborated by [43]. Although present throughout the whole transect, iSIS detected higher densities of the sea lily *Bathycrinus carpenterii* towards the last two thirds of the transect whereas the opposite was true for the sea cucumber *Kolga hyalina*. This could be a result of species interactions and/or differences in the spatial distribution of resources. Since megafaunal organisms affect the distribution of smaller-sized biota and shape benthic food webs through predation such findings are important in understanding ecosystem functioning. Furthermore, the envisaged application of iSIS to HG IV footage from different years will enable us to assess changes in the distribution of key megafaunal species over time in an area particularly vulnerable to the effects of climate change. We will also apply iSIS to images from other HAUSGARTEN stations and possibly other benthic locations. Advances in camera technology, associated with higher image resolution, will allow improved detection performances in the near future.

iSIS shows how computerized image analysis can assist in the inspection and monitoring of deep-sea benthos. The results resemble those produced manually by human experts, whilst greatly reducing human time commitment and removing the negative effects of observer fatigue. Further publications on automated detection approaches for benthic images are worthwhile to investigate non-easily accessible marine areas without contemporary intervention in the benthic system by sampling gears. The development of such automated systems is a new field of marine research, and allows the creation of new tools to improve the ongoing efforts to explore and understand the vast uncharted regions of the seafloor.

## References

[1] Thomson J, Weaver P (1987). Geology and geochemistry of abyssal plains..

[2] Carney RS (1997). Basing conservation policies for the deep-sea oor on current-diversity concepts: a consideration of rarity.. Biodiversity and Conservation.

[3] Gage JD (2004). Diversity in deep-sea benthic macrofauna: the importance of local ecology, the larger scale, history and the Antarctic.. Deep Sea Research Part II: Topical Studies in Oceanography.

[4] Schwinghamer P (1981). Characteristic size distributions of integral benthic communities.. Canadian Journal of Fisheries and Aquatic Sciences.

[5] Lampitt RS, Billett DSM, Rice AL (1986). Biomass of the invertebrate megabenthos from 500 to 4100 m in the northeast Atlantic Ocean.. Marine Biology.

[6] Christiansen B, Thiel H (1992). Deep-sea epibenthic megafauna of the northeast Atlantic: Abundance and biomass at three mid-oceanic locations estimated from photographic transects.. Rowe GT, Pariente V, editors, Deep-Sea Food Chains and the Global Carbon Cycle, Springer Netherlands, volume 360 of NATO *Science Series*.

[7] Piepenburg D, Chernova N, Dorrien C, Gutt J, Neyelov A (1996). Megabenthic communities in the waters around Svalbard.. Polar Biology.

[8] Grassle JF, Sanders HL, Hessler RR, Rowe GT, McLellan T (1975). Pattern and zonation: a study of the bathyal megafauna using the research submersible Alvin.. Deep-Sea Research and Oceanographic Abstracts.

[9] Rex MA (1981). Community structure in the deep-sea benthos.. Annual Review of Ecology and Systematics.

[10] Collie J, Escanero G, Valentine P (1997). Effects of bottom fishing on the benthic megafauna of georges bank.. Marine Ecology Progress Series.

[11] Kaiser M, Rogers S, Ellis J (1999). Importance of benthic habitat complexity for demersal fish assemblages. In: American Fisheries Society Symposium.. volume 22,.

[12] Soltwedel T, Vopel K (2001). Bacterial abundance and biomass in response to organismgenerated habitat heterogeneity in deep-sea sediments.. Marine Ecology Progress Series.

[13] Hasemann C, Soltwedel T (2006). Small-scale heterogeneity in the arctic deep sea: impact of small coldwater-sponges on the diversity of benthic nematode communities.. Rep Polar Mar Res.

[14] Quéric N, Soltwedel T (2007). Impact of small-scale biogenic sediment structures on bacterial distribution and activity in arctic deep-sea sediments.. Marine Ecology.

[15] Gray J (1981). Detecting pollution induced changes in communities using the log-normal distribution of individuals among species.. Marine Pollution Bulletin.

[16] Feder H, Pearson T (1988). The benthic ecology of loch linnhe and loch eil, a sea-loch system on the west coast of scotland. v. biology of the dominant soft-bottom epifauna and their interaction with the infauna.. Journal of experimental marine biology and ecology.

[17] Freire J (1996). Feeding ecology of liocarcinus depurator (decapoda: Portunidae) in the ria de arousa (galicia, north-west spain): effects of habitat, season and life history.. Marine Biology.

[18] Sarda R, Foreman K, Werme C, Valiela I (1998). The impact of epifaunal predation on the structure of macroinfaunal invertebrate communities of tidal saltmarsh creeks.. Estuarine, Coastal and Shelf Science.

[19] Gallucci F, Fonseca G, Soltwedel T (2008). Effects of megafauna exclusion on nematode assemblages at a deep-sea site.. Deep Sea Research Part I: Oceanographic Research Papers.

[20] Huettel M, Gust G (1992). Impact of bioroughness on interfacial solute exchange in permeable sediments.. Marine Ecology Progress Series 89: 253,.

[21] Smith K, Kaufmann R, Wakefield W (1993). Mobile megafaunal activity monitored with a timelapse camera in the abyssal north pacific.. Deep Sea Research Part I: Oceanographic Research Papers.

[22] Piepenburg D, Blackburn T, Dorrien C, Gutt J (1995). Partitioning of benthic community respiration in the arctic (northwestern barents sea).. Marine Ecology Progress Series.

[23] Bett B, Malzone M, Narayanaswamy B, Wigham B (2001). Temporal variability in phytodetritus and megabenthic activity at the seabed in the deep northeast Atlantic.. Progress in Oceanography.

[24] Lochte K, Pfannkuche O, Wefer G, Billett D, Hebbeln D (2003). Processes driven by the small sized organisms at the water-sediment interface..

[25] Guillén J, Soriano S, Demestre M, Falqués A, Palanques A (2008). Alteration of bottom roughness by benthic organisms in a sandy coastal environment.. Continental Shelf Research.

[26] Sumida P, Bernardino A, Stedall V, Glover A, Smith C (2008). Temporal changes in benthic megafaunal abundance and composition across the west antarctic peninsula shelf: results from video surveys.. Deep Sea Research Part II: Topical Studies in Oceanography.

[27] Ruhl H (2007). Abundance and size distribution dynamics of abyssal epibenthic megafauna in the northeast pacific.. Ecology.

[28] Kaufmann R, Smith K (1997). Activity patterns of mobile epibenthic megafauna at an abyssal site in the eastern North Pacific: Results from a 17-month time-lapse photographic study..

[29] Hartmut, Bluhm (2001). Re-establishment of an abyssal megabenthic community after experimental physical disturbance of the seaoor.. Deep Sea Research Part II: Topical Studies in Oceanography.

[30] Kogan I, Paull CK, Kuhnz LA, Burton EJ, Thun SV (2006). ATOC/Pioneer seamount cable after 8 years on the seaoor: Observations, environmental impact.. Continental Shelf Research.

[31] Bergmann M, Soltwedel T, Klages M (2011). The interannual variability of megafaunal assemblages in the arctic deep sea: Preliminary results from the HAUSGARTEN observatory (79 N).. Deep Sea Research Part I: Oceanographic Research Papers.

[32] Smith KL, Ruhl HA, Bett BJ, Billett DSM, Lampitt RS (2009). Climate, carbon cycling, and deep-ocean ecosystems.. Proceedings of The National Academy of Sciences.

[33] Billett D, Bett B, Reid W, Boorman B, Priede I (2010). Long-term change in the abyssal ne Atlantic: The Amperima Event revisited.. Deep Sea Research Part II: Topical Studies in Oceanography.

[34] Hunkins KL, Ewing M, Heezen BC, Menzies RJ (1960). Biological and geological observations on the first photographs of the Arctic Ocean deep-sea oor.. Limnology and Oceanography.

[35] Afanasev I (1978). Investigations of the deep-sea bottom fauna in the central part of the Arctic Ocean.. Oceanology.

[36] MacDonald IR, Bluhm BA, Iken K, Gagaev S, Strong S (2010). Benthic macrofauna and megafauna assemblages in the arctic deep-sea Canada Basin.. Deep Sea Research Part II: Topical Studies in Oceanography.

[37] Paul AZ, Menzies RJ (1974). Benthic ecology of the high arctic deep sea.. Marine Biology.

[38] Mayer M, Piepenburg D (1996). Epibenthic distribution patterns on the continental slope off East Greenland at 75 N.. Marine Ecology Progress Series.

[39] Starmans A, Gutt J (2002). Mega-epibenthic diversity: a polar comparison.. Marine Ecology Progress Series.

[40] Jones DOB, Bett BJ, Tyler PA (2007). Depth-related changes in the arctic epibenthic megafaunal assemblages of Kangerdlugssuaq, East Greenland.. Marine Biology Research.

[41] Schulz M, Bergmann M, von Juterzenka K, Soltwedel T (2010). Colonisation of hard substrata along a channel system in the deep Greenland Sea.. Polar Biology.

[42] Bluhm BA, MacDonald IR, Debenham C, Iken K (2005). Macro- and megabenthic communities in the high arctic Canada Basin: initial findings.. Polar Biology.

[43] Soltwedel T, Jaeckisch N, Ritter N, Hasemann C, Bergmann M (2009). Bathymetric patterns of megafaunal assemblages from the arctic deep-sea observatory HAUSGARTEN.. Deep Sea Research Part I: Oceanographic Research Papers.

[44] Soltwedel T, Bauerfeind E, Bergmann M, Budaeva N, Hoste E (2005). HAUSGARTEN: multidisciplinary investigations at a deep-sea, long-term observatory in the Arctic Ocean.. Oceanography.

[45] Frauenheim K, Neumann V, Thiel H, Türkay M (1989). The distribution of the larger epifauna during summer and winter in the North Sea and its suitability for environmental monitoring.. Senckenbergiana Maritima.

[46] van Leeuwen P, Rijnsdorp A, Vingerhoed B (1994). Variations in abundance and distribution of demersal fish species in the coastal zone of the southeastern North Sea between 1980 and 1993..

[47] Lindeboom H, De Groot S (1998). Impact-ii: The effects of different types of fisheries on the north sea and irish sea benthic ecosystems..

[48] Reiss H, Kröncke I, Ehrich S (2006). Estimating the catching efficiency of a 2-m beam trawl for sampling epifauna by removal experiments.. ICES Journal of Marine Science: Journal du Conseil.

[49] Hecker B (1994). Unusual megafaunal assemblages on the continental slope off Cape Hatteras.. Deep Sea Research Part II: Topical Studies in Oceanography.

[50] Nybakken J, Craig S, Smith-Beasley L, Moreno G, Summers A (1998). Distribution density and relative abundance of benthic invertebrate megafauna from three sites at the base of the continental slope off central California as determined by camera sled and beam trawl.. Deep Sea Research Part II: Topical Studies in Oceanography.

[51] Ruhl HA (2007). Abundance and size distribution dynamics of abyssal epibenthic megafauna in the northeast Pacific.. Ecology.

[52] Bergmann M, Langwald N, Ontrup J, Soltwedel T, Schewe I (2011). Megafaunal assemblages from two shelf stations west of Svalbard.. Marine Biology Research.

[53] Weaver ML, Noebe RD, Kaufmann MJ, Lauerman LML, Kaufmann RS (1996). Distribution and abundance of epibenthic megafauna at a long time-series station in the abyssal northeast Pacific.. Deep Sea Research Part I: Oceanographic Research Papers.

[54] Solan M, Germano JD, Rhoads DC, Smith C, Michaud E (2003). Towards a greater understanding of pattern, scale and process in marine benthic systems: a picture is worth a thousand worms.. Journal of Experimental Marine Biology and Ecology.

[55] Tyler P (2003). Ecosystems of the deep oceans, volume 28..

[56] Culverhouse P, Williams R, Reguera B, Herry V, Gonzlez-Gil S (2003). Do experts make mistakes? a comparison of human and machine identification of dinoagellates.. Marine Ecology Progress Series.

[57] MacLeod N, Benfield M, Culverhouse P (2010). Time to automate identification.. Nature.

[58] Roberts P, Jaffe J, Trivedi M (2009). A multiview, multimodal fusion framework for classifying small marine animals with an opto-acoustic imaging system.. Workshop on Applications of Computer Vision (WACV).

[59] Edgington D, Cline D, Davis D, Kerkez I, Mariette J (2006). Detecting, tracking and classifying animals in underwater video.. OCEANS 2006.

[60] Gordan M, Dancea O, Stoian I, Georgakis A, Tsatos O (2006). A new SVM-based architecture for object recognition in color underwater images with classification refinement by shape descriptors. In: IEEE International Conference on Automation, Quality and Testing.. volume 2,.

[61] Foresti G, Gentili S (2002). A hierarchical classification system for object recognition in underwater environments.. Oceanic Engineering, IEEE Journal of.

[62] Barat C, Rendas MJ (2006). A robust visual attention system for detecting manufactured objects in underwater video.. OCEANS 2006.

[63] Rova A, Mori G, Dill LM (2007). One fish, two fish, butterfish, trumpeter: Recognizing fish in underwater video.. IAPR Conference on Machine Vision Applications.

[64] Spampinato C, Giordano D, Di Salvo R, Chen-Burger YHJ, Fisher RB (2010). Automatic fish classification for underwater species behavior understanding.. http://doi.acm.org/10.1145/1877868.1877881.

[65] Di Gesu V, Isgro F, Tegolo D, Trucco E (2003). Finding essential features for tracking starfish in a video sequence.. Proceedings of the 12th International Conference on Image Analysis and Processing.

[66] Powell J, Krotosky S, Ochoa B, Checkley D, Cosman P (2003). Detection and identification of sardine eggs at sea using a machine vision system. In: OCEANS 2003. volume 1, p.. 175.

[67] Clement R, Dunbabin M, Wyeth G (2005). Toward robust image detection of crown-of-thorns starfish for autonomous population monitoring.. Australasian Conference on Robotics & Automation.

[68] Purser A, Bergmann M, Lundälv T, Ontrup J, Nattkemper TW (2009). Use of machine-learning algorithms for the automated detection of cold-water coral habitats - a pilot study..

[69] Rigby P, Pizarro O, Williams S (2010). Toward adaptive benthic habitat mapping using gaussian process classification.. Journal of Field Robotics.

[70] Taylor R, Vine N, York A, Lerner S, Hart D (2008). Evolution of a benthic imaging system from a towed camera to an automated habitat characterization system.. OCEANS 2008.

[71] York A, Gallager S, Taylor R, Vine N, Lerner S (2008). Using a towed optical habitat mapping system to monitor the invasive tunicate species Didemnum sp. along the northeast continental shelf.. OCEANS 2008.

[72] Howland J, Gallager S, Singh H, Girard A, Abrams L (2006). Development of a towed survey system for deployment by the fishing industry.. OCEANS 2006.

[73] Teixido N, Albajes-Eizagirre A, Bolbo D, Hir EL, Demestre M (2011). Hierarchical segmentation-based software for cover classification analyses of seabed images (seascape).. Mar Ecol Prog Ser.

[74] Premke K, Muyakshin S, Klages M, Wegner J (2003). Evidence for long-range chemoreceptive tracking of food odour in deep-sea scavengers by scanning sonar data.. Journal of Experimental Marine Biology and Ecology.

[75] Premke K, Klages M, Arntz W (2006). Aggregations of arctic deep-sea scavengers at large food falls: temporal distribution, consumption rates and population structure..

[76] Gallucci F, Sauter E, Sachs O, Klages M, Soltwedel T (2008). Caging experiment in the deep sea: Efficiency and artefacts from a case study at the arctic long-term observatory HAUSGARTEN.. Journal of Experimental Marine Biology and Ecology.

[77] Quric N, Arrieta, JM, Soltwedel T, Arntz W (2008). Characterization of prokaryotic community dynamics in the sedimentary microenvironment of the demosponge Tentorium semisuberites from arctic deep waters..

[78] Kanzog C, Ramette A (2009). Microbial colonisation of artificial and deep-sea sediments in the Arctic Ocean.. Marine Ecology.

[79] Kanzog C, Ramette A, Quric N, Klages M (2009). Response of benthic microbial communities to chitin enrichment: an in situ study in the deep Arctic Ocean.. Polar Biology.

[80] Guilini K, Van Oevelen D, Soetaert K, Middelburg J, Vanreusel A (2010). Nutritional importance of benthic bacteria for deep-sea nematodes from the arctic ice margin: Results of an isotope tracer experiment.. Limnology and Oceanography.

[81] van Oevelen D, Bergmann M, Soetaert K, Bauerfeind E, Hasemann C (2011). Carbon ows in the benthic food web at the deep-sea observatory HAUSGARTEN (Fram Strait).. Deep Sea Research.

[82] Ontrup J, Ehnert N, Bergmann M, Nattkemper T (2009). BIIGLE - Web 2.0 enabled labelling and exploring of images from the Arctic deep-sea observatory HAUSGARTEN.. OCEANS 2009 - EUROPE.

[83] White OR (2006). http://www.docstoc.com/docs/72106981/Interobserver-Agreements.

[84] Sikora T (2001). The MPEG-7 visual standard for content description-an overview.. IEEE Transactions on Circuits and Systems for Video Technology.

[85] Baştan M, Çam H, Güdükbay U, Özgür Ulusoy (2010). BilVideo-7: An MPEG-7-compatible video indexing and retrieval system.. IEEE MultiMedia.

[86] Chen YQ, Nixon MS, Thomas DW (1995). Statistical geometrical features for texture classification.. Pattern Recognition.

[87] Daugman J (1988). Complete discrete 2-D Gabor transforms by neural networks for image analysis and compression.. Acoustics, Speech and Signal Processing, IEEE Transactions on.

[88] Ontrup J, Wersing H, Ritter H (2004). A computational feature binding model of human texture perception.. Cognitive Processing.

[89] Caliski T, Harabasz J (1974). A dendrite method for cluster analysis.. Communications in Statistics.

[90] Bandyopadhyay S, Maulik U, Mukhopadhyay A (2007). Multiobjective genetic clustering for pixel classification in remote sensing imagery.. Geoscience and Remote Sensing, IEEE Transactions on.

[91] Davies DL, Bouldin DW (1979). A cluster separation measure.. Pattern Analysis and Machine Intelligence, IEEE Transactions on.

[92] Rosenfeld A, Kak AC (1982). Digital Picture Processing..

[93] Vapnik V (2000). The nature of statistical learning theory..

[94] Cristianini N, Shawe-Taylor J (2000). An introduction to support vector machines: and other kernel-based learning methods..

[95] Joachims T (1999). Making large-scale SVM learning practical.. Advances in Kernel Methods - Support Vector Learning, Cambridge, MA: MIT Press, chapter 11.

[96] Bishop CM (2007). Pattern Recognition and Machine Learning (Information Science and Statistics)..

[97] Platt JC (1999). Probabilistic outputs for support vector machines and comparisons to regularized likelihood methods. In: Advances in large margin classifiers.. MIT Press,.

